# Feeling of pulsations in artificial arteries with a real time haptic feedback laparoscopic grasper: a validation study

**DOI:** 10.1007/s00464-024-10877-w

**Published:** 2024-06-10

**Authors:** Michel P. H. Vleugels, Masie Rahimi

**Affiliations:** 1 Department of Obstetrics and Gynecology, Hospital Clinica Benidorm, Benidorm, Spain; 2 Department of Surgery, Amsterdam UMC – VU University Medical Center, Amsterdam, The Netherlands; 3 Amsterdam Skills Centre for Health Sciences, Amsterdam, The Netherlands; 4 Cancer Center Amsterdam, Amsterdam, The Netherlands; 5 Malden, The Netherlands

**Keywords:** Laparoscopic surgery, Arterial pulsations, Realtime haptic feedback

## Abstract

**Introduction:**

Despite the advancements in technology and organized training for surgeons in laparoscopic surgery, the persistent challenge of not being able to feel the resistance and characteristics of the tissue, including pulsations, remains unmet. A recently developed grasper (Optigrip®) with real time haptic feedback, based on photonic technology, aims to address this issue by restoring the tactile sensation for surgeons. The key question is whether pulsations can be detected and at what minimal size level they become clinical significant.

**Methods:**

To simulate arterial conditions during laparoscopic procedures, four different silicone tubes were created, representing the most prevalent arteries. These tubes were connected to a validated pressure system, generating a natural pulse ranging between 80 and 120 mm Hg. One control tube without pressure was added. The surgeons had to grasp these tubes blindly with the conventional grasper or the haptic feedback grasper in a randomized order. They then indicated whether they felt the pressure or not and the percentage of correct answers was calculated.

**Results:**

The haptic grasper successfully detected 96% of all pulsations, while the conventional grasper could only detect 6%. When considering the size of the arteries, the Optigrip® identified pulsations in 100% the 4 and 5 mm arteries and 92% of the smallest arteries. The conventional grasper was only able to feel the smallest arteries in 8%. These differences were highly significant (*p* < 0.0001).

**Conclusion:**

This study demonstrated that the newly developed haptic feedback grasper enables detection of arterial pulsations during laparoscopy, filling an important absence in tactile perception within laparoscopic surgery.

Laparoscopic surgery has become the standard for most of abdominal procedures, including intestinal surgery, urological and gynecological interventions [1–6]. Initially, it was a challenge for the performing surgeon to perform the first laparoscopic procedures due to the known disadvantages. The lack of depth perception, mirrored movements, the angle between the hand and the image, limited self-control on the viewpoint, and the non-ergonomic graspers are known challenges for the surgeon [7–11]. Some of these issues have been addressed by new technologies or robotic systems [7–12].

The unresolved challenge lies in the reduced feeling of the resistance and characteristics of the grasped tissue, which is five times less than that of the hand [13, 14]. This lack of haptic feedback can be compensated partially by the visual feedback of tissue and instrument interaction after a long learning curve [12, 15, 16]. Observing the tissue’s movement in the beak of the grasper can guide the surgeon in adjusting the beak, preventing unexpected damage to vulnerable tissue. However, visual feedback alone does not improve the performance of the surgeon as visual feedback and haptic feedback does [17].

Due to the absence of haptic feedback pulsation of arteries cannot be felt [14]. Often, the pulsations of a bigger artery can be seen. However, in many surgical conditions it is difficult to distinguish arteries from other structures due to fibrotic tissue, infectious tissue or changed anatomy [18, 19].

During laparoscopic procedures, it is important to recognize the arterial vessels to prevent damage and to be able to ligate the artery in preparation of a procedure. Feeling the pulsation of the artery will speed up the preparation phase and creates the confidence that the artery even without completely being prepared can be ligated or spared.

The aim of this study was to investigate whether real time haptic feedback in a newly designed laparoscopic grasper (Optigrip®) is able to detect arterial pulsations better than a conventional laparoscopic grasper.

## Methods

### Laparoscopic haptic grasper

**Fig. 1:**
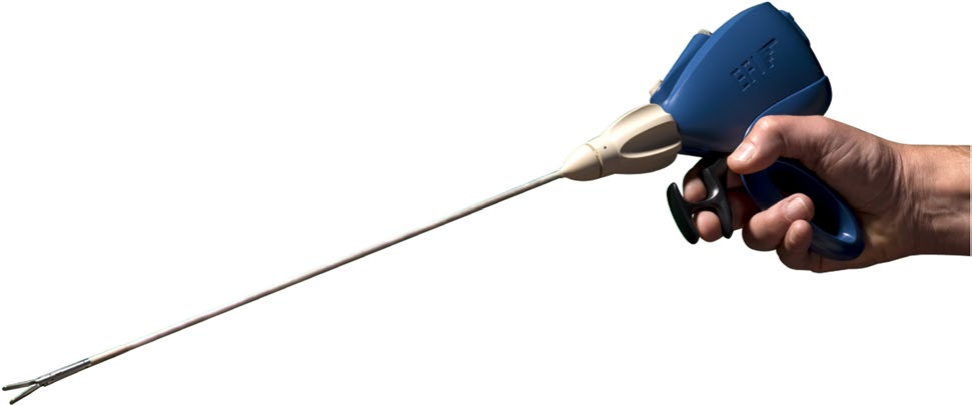
The Optigrip grasper

The Optigrip® is a reusable laparoscopic grasper as usual with a pistol grip. The shaft is inserted into the handpiece with the pistol grip and is interchangeable. The specific tissue resistance which is felt by the grasping beak creates a tension on a fiber Bragg (5) sensor on a glass fiber in the beak (Fig. 1). This tension creates a shift of the reflected light frequencies by the fiber Bragg gratings on the level of nanometers caused by the compressing or stretching of these gratings (6, 7). This light frequency change of the light reflection is measured 6000 pro second in the control box Optigator which translates this in an activation of the actuator in the handgrip. The specific tissue resistance is felt as resistance on the gripper once the surgeon closes the beak. Not only the resistance of the tissue is felt grasping the tissue by closing the beak but also the tissue resistance against the beak from outside. This is important during preparing tissue and dividing tissue layers.

To optimize the feeling of minimal tissue differences on the fingertips a pistol grip was developed as a pistol grip is the most ergonomic gripper which also is preferred by the most surgeons (8). This study will compare the Optigrip® and a conventional laparoscopic grasper in feeling of arterial pulsations.

### Artificial vessels characteristics

**Fig. 2:**
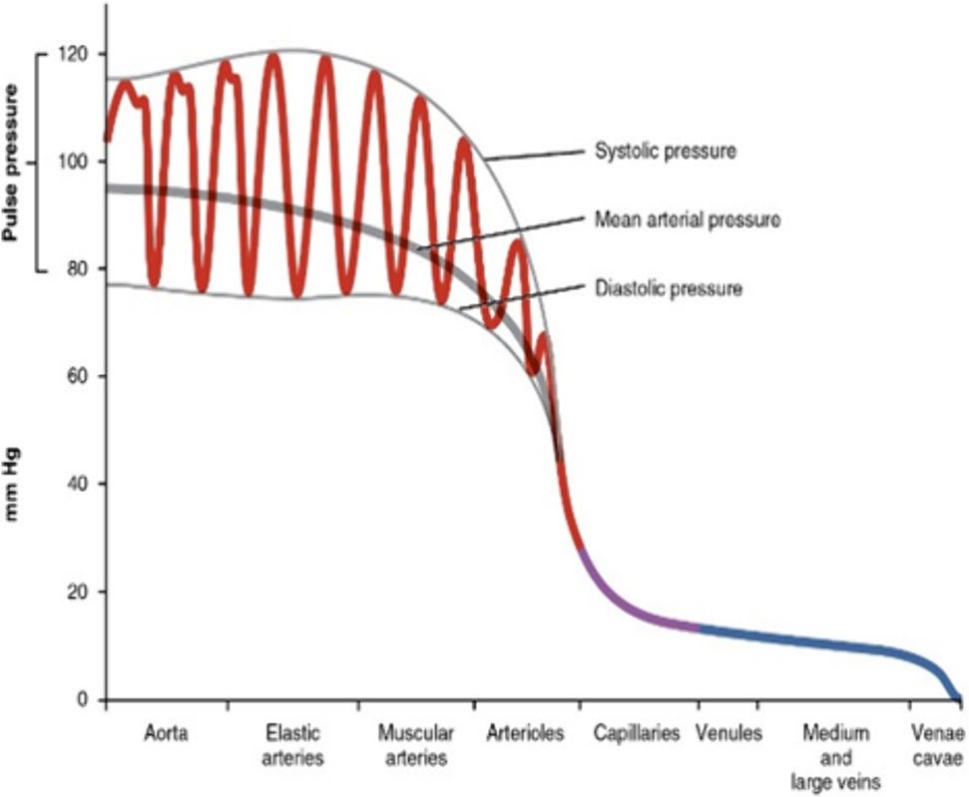
Pulse difference related to vessel type [20]

**Fig. 3:**
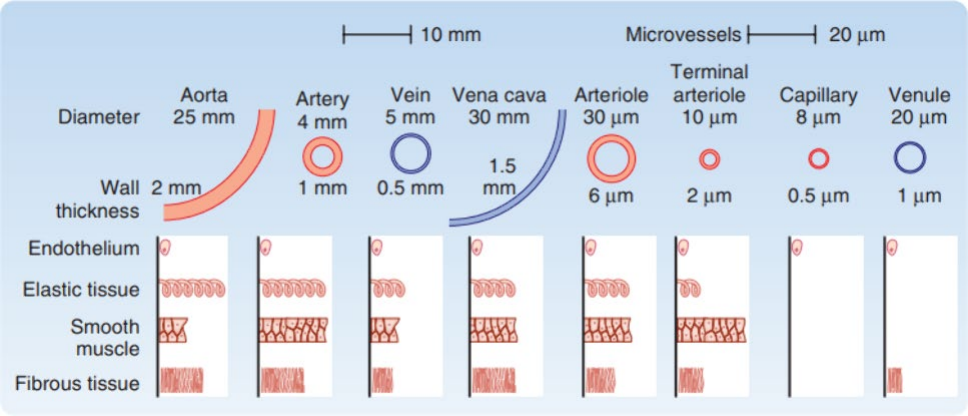
Diameter and wall thickness of vessels [21]

The pulse which can be sensed by touch during laparotomy is defined by the pressure difference between the diastolic and systolic pressure (Fig. 2). This pressure difference is more evident in arterial vessels and not in the veins REF. This pressure difference varies during the day, depending on our activity, the size of the artery and the specific wall thickness of the arteries (Figs. 2, and 3) [20]. The diameters of the critical arteries in minimal invasive surgery are small, with outer diameter varying between 1 and 8 mm (Fig. 3). The wall thickness of the arteries varies from 1 to 1.5 mm. (Fig. 3) [20, 21].

To define which arteries are of clinical importance in laparoscopic surgery, arteries were selected that are of clinical importance during surgery for the following medical specialties: urology, gynecology, and intestinal surgery. These arteries have been described (Table 1) and depending on their outer diameter and thickness of the wall divided into 4 groups (Table 2). To mimic these arteries, four silicone tubes have been made representing these four different groups of diameters. These tubes represent the average outer diameter, the wall thickness, and the inner diameter of these four categories.

**Table 1:** Relevant artery diameters and tube categories

	Outer diameter (mm)	Tube test category
Gastro-intestinal surgeon		
Splenic artery	5–8	D
Superior mesenteric artery	6–8	D
External iliac artery	6–8	D
Inferior mesenteric artery	3–5	C
Gastric artery left/right	2–4	B
Colic arteries left, right, middle	2–4	B
Superior pancreaticoduodenal artery	2–4	B
Intestinal arteries	1–3	A
Urological surgeon		
Renal artery	5–7	D
Vesical artery	2–4	B
Testicular artery	1–3	A
Gynaecological surgeon		
Internal iliac artery	4–6	C
Uterine artery	2–4	B
Ovarian artery	1–3	A
Thoracic surgeon		
Pulmonal artery	5–6	D
Bronchial artery	1–3	A

**Table 2:** Four categories of artificial vessel sizes representing the four groups of arteries

	Inner diameter (mm)	Outer diameter (mm)	Wall thickness (mm)
A	1.4	2	0.3
B	2	2.6	0.3
C	3	3.8	0.4
D	4	5	0.5

### Laparoscopy setup

**Fig. 4:**
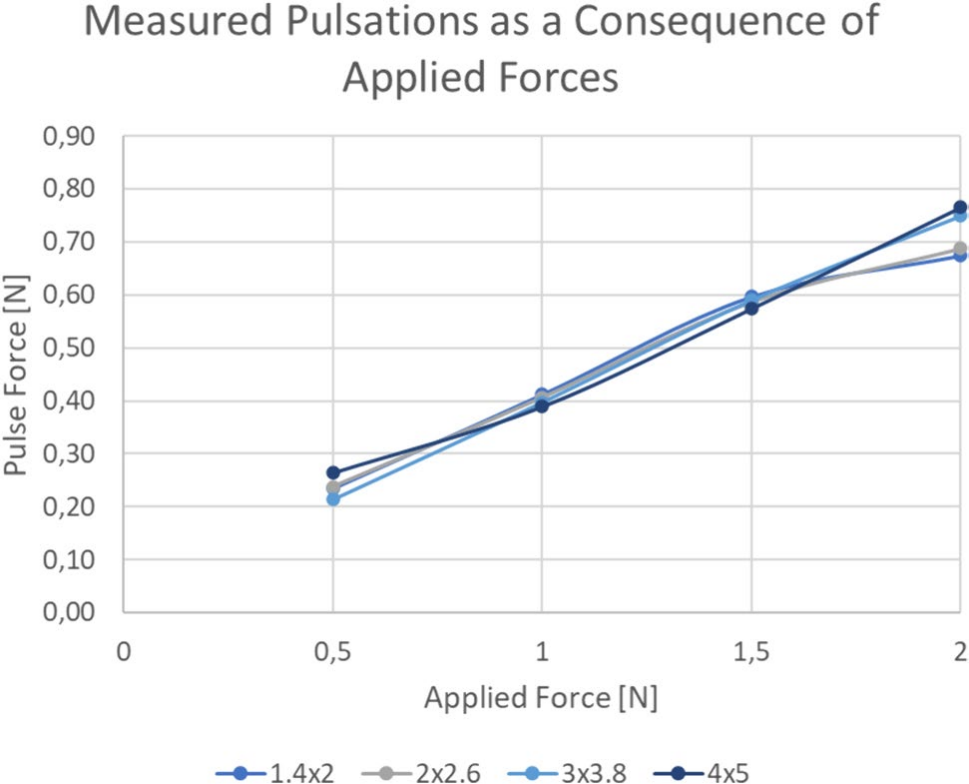
Linear relation between force and pulsation in all four artificial blood vessels

A pressure pump created a systolic pressure of 120 mmHg. To control whether the pressure and so the pulse is stable and equal all over the length of these tubes, the relation between the applied force and pressure was tested. There appeared to be a linear relation between the force and the pressure for all the four tube sizes. (Fig. 4).

**Fig. 5:**
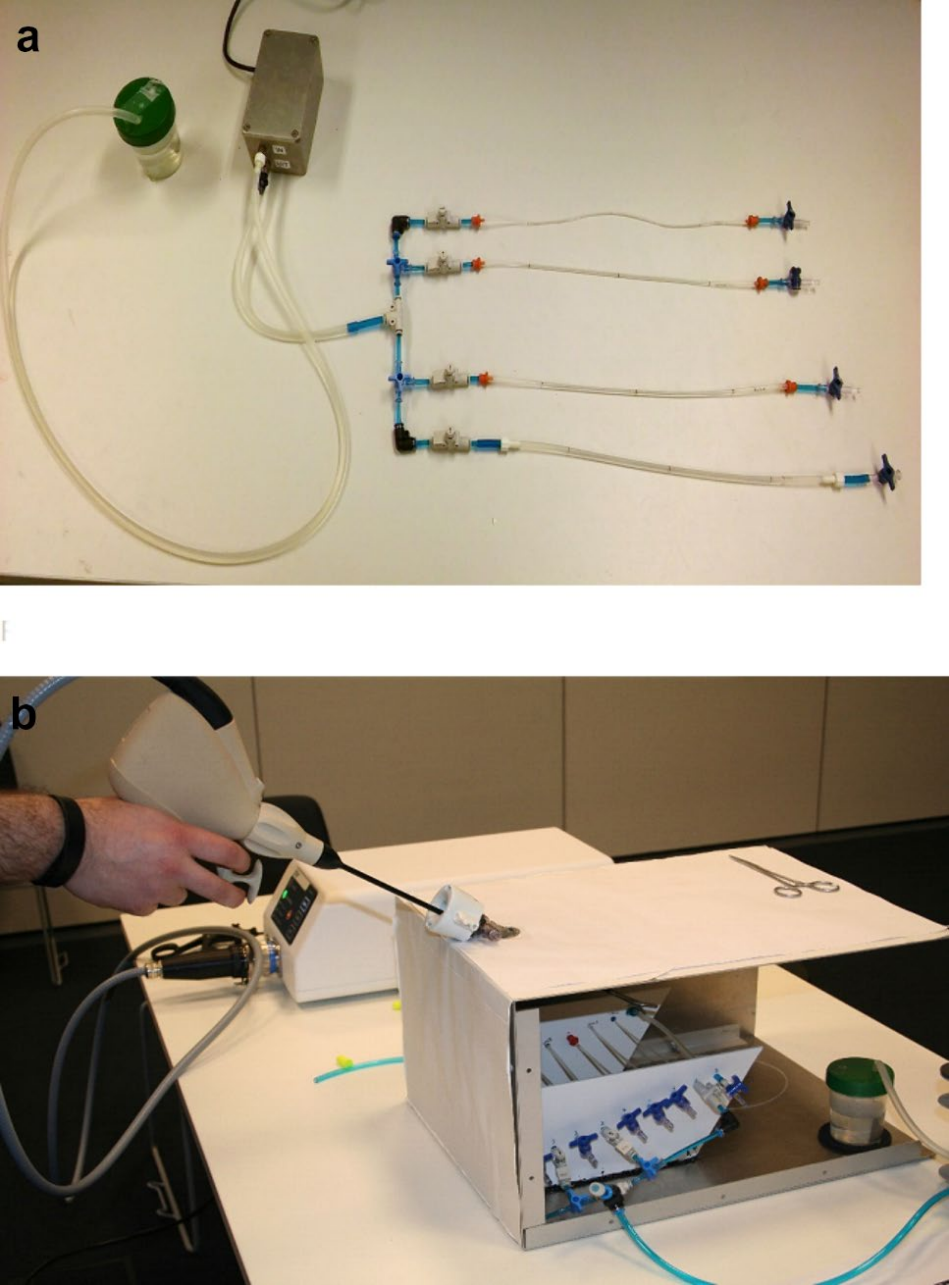
**a** Pump and four prepared tubes. **b** Black box trainer with the 5 tubes inside

A laparoscopic box trainer was created in which the four artificial blood vessels were fixed. Also, a control tube without pulse was added (Fig. 5a and b).

### Protocol

This study was conducted in the simulation lab of the Amsterdam Skills Centre (ASC, Amsterdam, The Netherlands). A randomized controlled trial study design was incorporated. Surgical residents (PGY 3–4) from Dutch teaching hospitals were included. IRB approval was acquired from the Amsterdam UMC and all participants provided written informed consent. A laparoscopic pulse sense test was performed using a black laparoscopic box trainer. Participants had to grasp the 5 tubes in a randomized sequence and they were asked whether they could feel the pulse. An assistant guided the grasper to the artificial vessels and the participant was allowed to grasp the tube for 3 s. The pulse sense test was performed using the haptic feedback grasper and a conventional laparoscopic grasper (KELLY grasping forceps, Karl Storz SE & Co. KG, Tuttlingen, Germany). A conventional laparoscopic curved dissection forceps was chosen due to its common usage in laparoscopic procedures for tissue manipulation and dissection, ensuring a realistic control.

The sequence of the use of these two graspers was done in a randomized order. The participants were blinded to the size of the tubes. After this grasping action they were asked whether they could feel the pulse yes or no. Once they grasped all five tubes with one grasper they repeated the procedure with the other grasper.

### Statistical analyses

The results were scored as the number and percentage of correct answers. The percentages were calculated by adding all correct answers for every tool and comparing this to the maximum possible score for every tool (5 answers * 12 = 60.) It was expected that both graspers would at least have 20% of the answers correct as there was one non-pulsating control tube out of the total of five tubes. Based on a prior pilot study a post hoc power analysis was performed to determine the group size. Based on a power of 0.80 and a significance of 0.05 the sample size consisted of *n* = 11. These results were analyzed with the Wilcoxon signed rank test. Individual tube diameter and grasper were also compared using the Fisher’s Exact Test. Values of *p* < 0.05 were considered statistically significant.

## Results

**Fig. 6:**
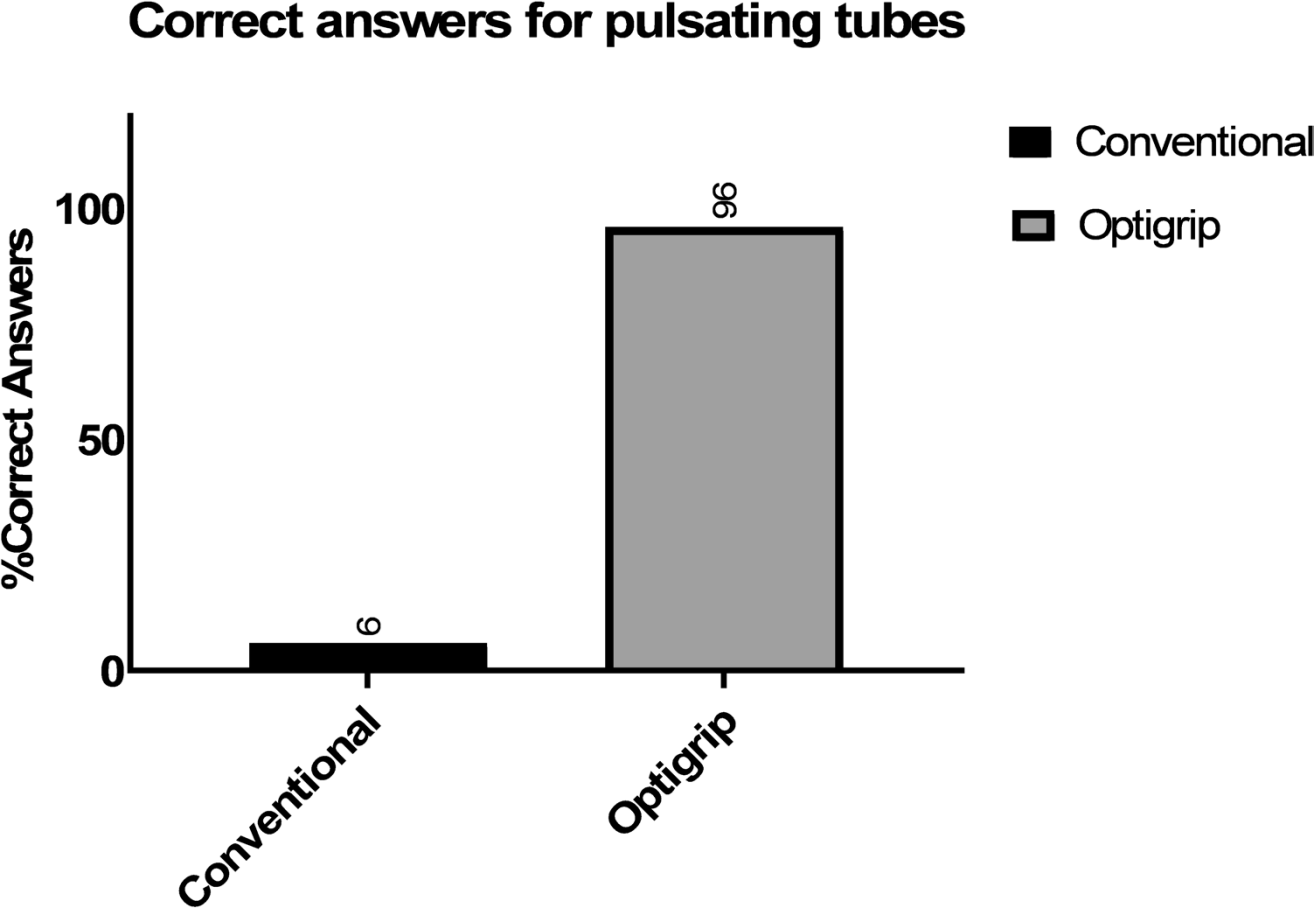
Percentage of correct answers for only pulsating tubes

**Fig. 7:**
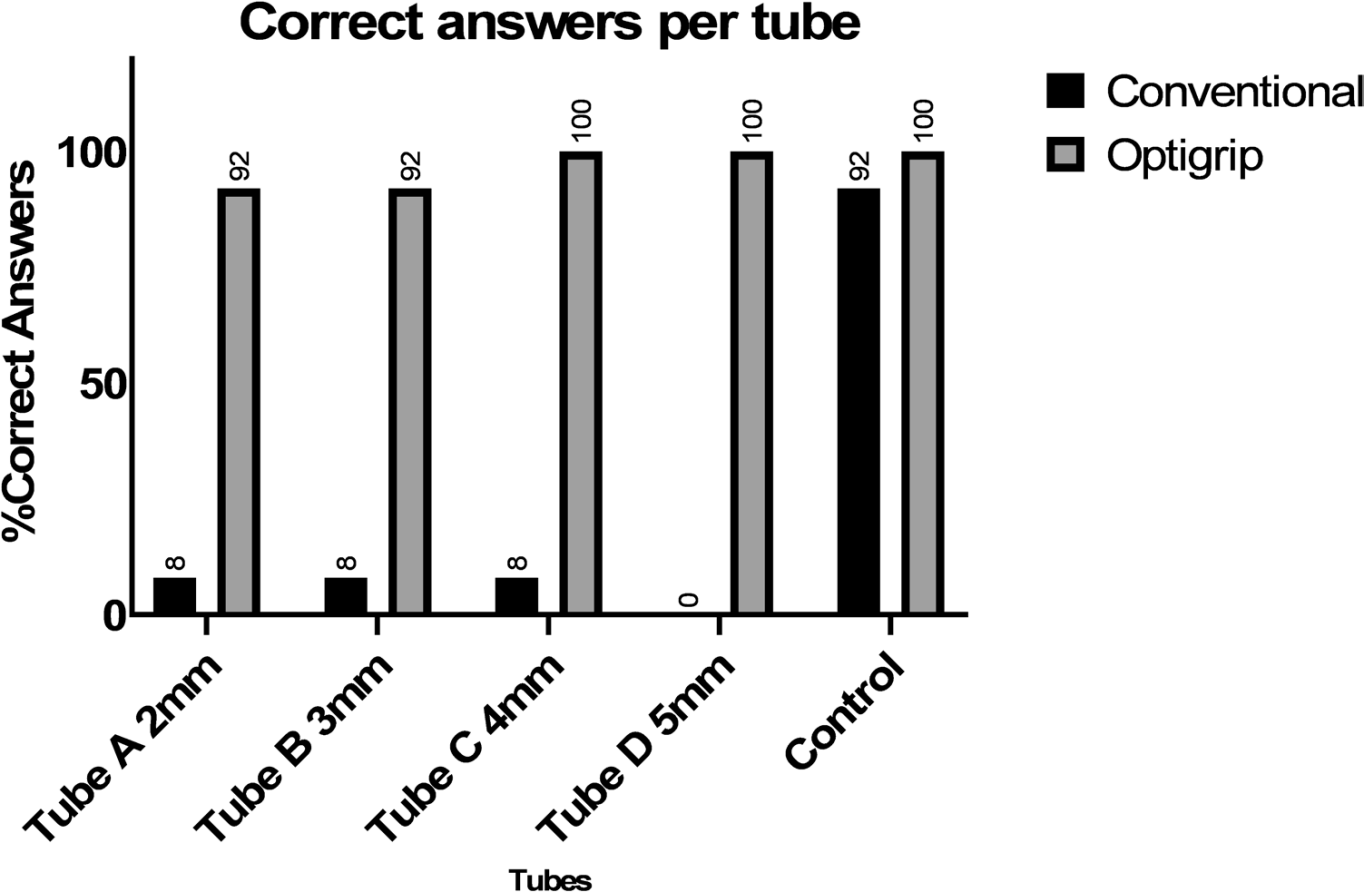
Correct answers according tubes

A total of 12 surgical residents participated in the study. The percentage of correct answers of all pulsating tubes for both types of graspers, conventional and the haptic feedback grasper are presented (Figs. 6 and 7).

With the haptic feedback grasper subjects correctly identified pulsating tubes of all assessments (96%), compared to 6% correct answers with the conventional grasper. The Optigrip® enabled more accurate detection of laparoscopic artery pulsations for Tube A 2 mm (8% vs 92%), Tube B 3 mm (8% vs 92%), Tube C 4 mm (8% vs 100%), Tube D 5 mm (0% vs 100%) and the control tube (92% vs 100%), compared to the conventional grasper. The percentage of corrects answers for both graspers for each of the tube diameters and the non-pulsating tube are shown in Fig. 7. The difference between the Optigrip® and the conventional grasper was significantly different (*p* < 0.001: Wilcoxon signed rank test).

For each individual diameter, the Optigrip® was also more accurate for all tubes: Tube A 2 mm (Conventional 1 correct and Haptic Grasper 11 correct *p* < 0.001), Tube B 3 mm (Conventional 1 correct and Haptic Grasper 11 correct *p* < 0.001), Tube C 4 mm (Conventional 1 correct and Haptic Grasper 12 correct *p* < 0.001) and Tube D 5 mm (Conventional 0 correct and Haptic Grasper 12 correct *p* < 0.001) (Table 3).

**Table 3:** Fisher’s Exact Test for each tube

	Conventional Correct	Conventional Incorrect	Haptic Grasper Correct	Haptic Grasper Incorrect	Significance
Tube A (2 mm)	1	11	11	1	*p* < 0.001
Tube B (3 mm)	1	11	11	1	*p* < 0.001
Tube C (4 mm)	1	11	12	0	*p* < 0.001
Tube D (5 mm)	0	12	12	0	*p* < 0.001

## Discussion

In this study, we investigated whether arterial pulsations can be felt with a newly designed real time haptic feedback grasper, (Optigrip®). We compared this new grasper with a conventional grasper. To copy the daily practice of laparoscopy, we selected all arteries which we encounter during laparoscopy and divided these into 4 groups. The average size of the inner and outer diameter of these 4 groups have been copied to these 4 artificial arteries. Also, a pulsating system has been developed to mimic the arterial pressure wave.

These results demonstrated that the Optigrip®, equipped with real time haptic feedback, could detect arterial pulsations in 96% of the artificial arteries, whereas the conventional grasper achieved this in only 6%. The ability to feel pulsations remained consistent across all four different diameters of the arteries. Notably, participants using the conventional grasper failed to feel pulsations in the 4 mm and 5 mm tubes, raising the question about the potential for detection pulsations in 2 mm and 3 mm tubes.

Laparoscopic interventions have become the standard for most of the abdominal operations, intestinal surgery as well as for urological or gynecological interventions. Initially it was a challenge for the surgeon to perform the first laparoscopic procedures due to the known challenges, like the mirrored movements, lack of good clear vision or three-dimensional vision and non-ergonomic instruments [22]. Most of these disadvantages of laparoscopic surgery for the surgeon have been solved, like High-Definition screens, three-dimensional systems, ergonomic handgrips At last but not at least the reduced feeling of the resistance and characteristics of the grasped tissue by the conventional grasper, which is 5 times less than de hand in conventional laparoscopic surgery (1) and absent in robotic surgery (12) is still an unmet need of the surgeon [13].

This lack of this haptic feedback can be compensated partially by the visual haptic feedback after long learning curve, [15] the consequence of this reduced feeling of the touch of the tissue, is that the surgeon uses more squeezing force of the grasper as is necessary to manipulate the tissue [23]. By creating the haptic feedback in the grasper, the surgeon automatically reduces his grasping force in his handle [24]. In most cases, surgeons pay extra attention to vulnerable tissue with regards to the grasping force. However, when the anatomy has been changed due to infection or fibrosis. Moreover, this might be of importance during the initial learning curve of surgeons in laparoscopic surgery. By reducing this squeezing force, a reduction of complications due to unexpected damage of bowel and vulnerable organs might be expected with a reduction of surgical costs [25].

The ultimate feeling of our fingers is the pulsations of small arteries. This feeling is lost completely with the surgical laparoscopic graspers. This new fiber-optical system creates a real time haptic feedback grasper with a loop of 6000 pro second which makes it possible to feel small arterial pulsations. This study has proven that the haptic feedback grasper restores this lack of information completely compared to the conventional grasper. Haptic feedback is absent in robotic surgery [26, 27]. The use of this technology could be of significant interest to overcome this drawback of robotic surgery [28]. In this study we selected only those arteries which are of importance in abdominal-urological-gynecological surgery. This haptic feedback might be even more important for the thoracic-vascular surgeon as well [29].

Often, the pulsations of a bigger artery can be seen. However, in many surgical conditions it is difficult to distinguish the smallest arteries from other structures due to fibrotic tissue, infectious tissue or changed anatomy. By feeling those pulsating structures, manipulating and preparing the structures free from the surrounding tissues will be done with more confidence and quicker as has been described by a dozen of surgeons who operated with this new grasper [30].

## Conclusion

This study demonstrated that the newly developed haptic feedback grasper enables detection of arterial pulsations during laparoscopy, filling an important absence in tactile perception within laparoscopic surgery. An important potential of this technology is the implementation in robotic assisted surgery.
